# The HIF-1α/PLOD2 axis integrates extracellular matrix organization and cell metabolism leading to aberrant musculoskeletal repair

**DOI:** 10.1038/s41413-024-00320-0

**Published:** 2024-03-12

**Authors:** Heeseog Kang, Amy L. Strong, Yuxiao Sun, Lei Guo, Conan Juan, Alec C. Bancroft, Ji Hae Choi, Chase A. Pagani, Aysel A. Fernandes, Michael Woodard, Juhoon Lee, Sowmya Ramesh, Aaron W. James, David Hudson, Kevin N. Dalby, Lin Xu, Robert J. Tower, Benjamin Levi

**Affiliations:** 1 Center for Organogenesis, Regeneration and Trauma, Department of Surgery, University of Texas Southwestern, Dallas, TX 75390 USA; 2 Section of Plastic Surgery, Department of Surgery, University of Michigan, Ann Arbor, MI 48109 USA; 3 Quantitative Biomedical Research Center, Peter O’Donnell Jr. School of Public Health, University of Texas Southwestern, Dallas, TX 75390 USA; 4 Department of Orthopedics and Sports Medicine, University of Washington, Seattle, WA 98195 USA; 5 Division of Chemical Biology and Medicinal Chemistry, College of Pharmacy, University of Texas at Austin, Austin, TX 78712 USA; 6 Department of Pathology, Johns Hopkins University, Baltimore, MD 21218 USA

**Keywords:** Pathogenesis, Bone

## Abstract

While hypoxic signaling has been shown to play a role in many cellular processes, its role in metabolism-linked extracellular matrix (ECM) organization and downstream processes of cell fate after musculoskeletal injury remains to be determined. Heterotopic ossification (HO) is a debilitating condition where abnormal bone formation occurs within extra-skeletal tissues. Hypoxia and hypoxia-inducible factor 1α (HIF-1α) activation have been shown to promote HO. However, the underlying molecular mechanisms by which the HIF-1α pathway in mesenchymal progenitor cells (MPCs) contributes to pathologic bone formation remain to be elucidated. Here, we used a proven mouse injury-induced HO model to investigate the role of HIF-1α on aberrant cell fate. Using single-cell RNA sequencing (scRNA-seq) and spatial transcriptomics analyses of the HO site, we found that collagen ECM organization is the most highly up-regulated biological process in MPCs. Zeugopod mesenchymal cell-specific deletion of *Hif1α* (*Hoxa11-CreER*^*T2*^; *Hif1a*^*fl/fl*^) significantly mitigated HO in vivo. ScRNA-seq analysis of these *Hoxa11-CreER*^*T2*^; *Hif1a*^*fl/fl*^ mice identified the PLOD2/LOX pathway for collagen cross-linking as downstream of the HIF-1α regulation of HO. Importantly, our scRNA-seq data and mechanistic studies further uncovered that glucose metabolism in MPCs is most highly impacted by HIF-1α deletion. From a translational aspect, a pan-LOX inhibitor significantly decreased HO. A newly screened compound revealed that the inhibition of PLOD2 activity in MPCs significantly decreased osteogenic differentiation and glycolytic metabolism. This suggests that the HIF-1α/PLOD2/LOX axis linked to metabolism regulates HO-forming MPC fate. These results suggest that the HIF-1α/PLOD2/LOX pathway represents a promising strategy to mitigate HO formation.

## Introduction

Musculoskeletal injury and postsurgical changes can cause the pathologic formation of cartilage and bone outside of the native skeleton in a process called heterotopic ossification (HO).^1^ We and others have shown that this process occurs due to the aberrant differentiation of local mesenchymal progenitor cells (MPCs), which inappropriately form bone through intramembranous or endochondral ossification.^2,3^ Hypoxia is frequently induced during surgeries with a higher risk for HO with the routine use of tourniquets to minimize blood loss. Additionally, patients are surviving previously un-survivable injuries with increased limb salvage after extremity trauma due to the more widespread use of tourniquets. With this increased survival and limb salvage secondary to tourniquet use, there is a resultant rise in wound hypoxia.^4^ Hypoxia-inducible factors (HIFs) are heterodimeric transcription factors consisting of an oxygen-sensitive alpha subunit (HIF-1α, HIF-2α, or HIF-3α in mammals) and a stable constitutively expressed beta subunit (HIF-1β). HIF-1α is expressed in all cell types, whereas HIF-2α expression is restricted to specific cell types, including vascular endothelial cells.^5^ HIF-1α is a significant mediator of cellular adaptation to hypoxia and pre-HO chondrogenesis.^6^ Under hypoxic conditions, stabilized HIF-1α translocates to the nucleus, dimerizes with HIF-1β, and binds to hypoxia response elements present within or near HIF-1-regulated genes. HIF-1α signaling is key to sustaining the differentiation of prechondrogenic cells within hypoxic conditions during skeletogenesis by regulating sex-determining region Y-box 9 (SOX-9),^7^ playing a critical role in normal muscle and cartilage formation during limb development.^8^ Using a genomic database of 244 burn patients at high risk for HO compared with unburned “control” patients, we previously found a significant elevation of *HIF1A* transcripts and activation of HIF-1α target genes including *vWF, PECAM, CDH5*, and *VEGFA*.^9^

Beyond osteochondral tissue and vascular development, HIF-1α has been implicated in ECM organization and remodeling.^10^ Type I collagen is the main component of the ECM in tendon and is synthesized in the endoplasmic reticulum as a procollagen undergoing extensive post-translational modifications.^11^ Hydroxylation of specific proline and lysine residues of procollagen is catalyzed by prolyl 4-hydroxylase (P4H) and procollagen-lysine, 2-oxoglutarate 5-dioxygenase (PLOD), respectively. Among three isoforms (PLOD1, PLOD2, and PLOD3) in mammalian cells, PLOD2 specifically hydroxylates lysine residues in the telopeptide of procollagens.^12^ After procollagen secretion, collagen cross-linking by lysyl oxidase (LOX) family enzymes (LOX and LOXL1–LOXL4) occurs by the specific oxidative deamination of lysine or hydroxylysine residues in the telopeptide.^13^ Since cross-linking involving hydroxylysine residues is more stable than that involving lysine residues,^14^ PLOD2 plays a critical role in the stability of collagen cross-links and the formation of normal mature collagen. An increase in collagen cross-linking is associated with enhanced ECM stiffness, which has been shown to facilitate cell adhesion, migration, growth, and survival.^15^ The roles of HIF-1α signaling on the tumor collagen microenvironment and metabolic reprogramming have been well-defined.^16,17^ However, a fundamental understanding of how HIF-1α signaling modulates ECM and abnormal mesenchymal cell fate decisions after musculoskeletal injury is lacking. Recent work by our team and others have implicated the role of ECM organization on MPC fate.^18,19^ Given these basic and clinical observations, we hypothesized that cellular hypoxia signaling through HIF-1α would alter key cellular metabolism and ECM organization programs responsible for traumatic HO formation and progression.

Here, we sought to define the regulatory role of HIF-1α in our previously validated traumatic HO model.^20^ During HO formation and progression, we noted an increase in HIF-1α signaling primarily in MPCs using scRNA-seq, snATAC-seq, and spatial transcriptomic analyses of an HO injury. Lineage-specific HIF-1α deletion in MPCs restricted to the zeugopod in adulthood inhibited HO formation. To explain these observations, we analyzed HIF-1α and metabolic signaling at the HO site and noted a concurrent increase in collagen cross-linking enzymes, including PLOD2 and LOX family members (LOX, LOXL1, and LOXL2). Subsequently, we found that MPC-specific HIF-1α deletion decreased the upregulation of glycolytic rewiring and collagen fibril organization. These changes in signaling led to an alteration in collagen matrix alignment as mice with HIF-1α deletion in MPCs exhibited decreased ECM alignment, which we have shown can mitigate MPC osteogenic fate differentiation.^18,19^ In summary, we demonstrated that hypoxia induces HIF-1α signaling, which drives increased glycolysis thereby altering collagen cross-linking enzyme production and ECM alignment; this in turn alters MPC fate. These findings suggest that the HIF-1α/PLOD2 axis may represent a therapeutic target to mitigate aberrant MPC differentiation and HO.

## Results

### Zeugopod-specific HIF-1α deletion mitigates burn/tenotomy (BT)-induced HO

**Fig. 1:**
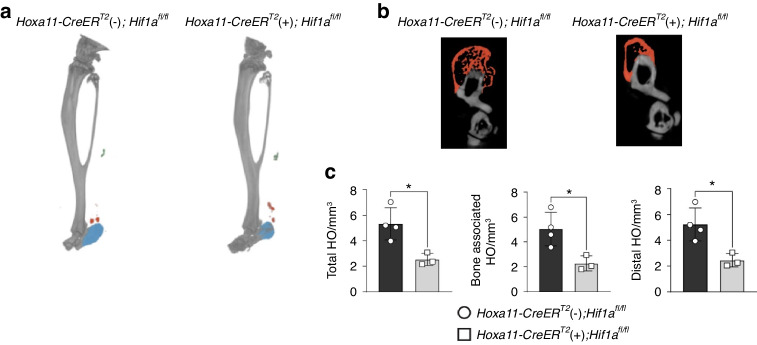
Zeugopod-specific HIF-1α deletion mitigates BT-induced HO. **a** (Upper panel) Representative 3-D reconstruction µCT images of BT injured limbs from *Hoxa11*-*CreER*^T2^ (−); *Hif1a*^fl/fl^ and *Hoxa11*-*CreER*^T2^ (+); *Hif1a*^fl/fl^ mice 9-weeks post-BT. Ectopic bone formation was indicated by pseudo-coloring the bone (blue, orange, and green). (Lower panel) Cross-sectional view of calcaneus bone-associated HO (orange). **b** Quantification of total, bone-associated, and distal HO. Error bars represent mean ± SD. *P < 0.05 vs. *Hoxa11*-*CreER*^T2^ (−); *Hif1a*^*fl/fl*^ mice. Mann-Whitney unpaired *t* test, two-tailed (*n* = 3–4/group)

*Hoxa11* is an embryonic patterning gene, of which expression is highest in the zeugopod during embryonic development.^21–23^ Using the *Hoxa11*-*CreER*^T2^; tdTom lineage tracing system and scRNA-seq, we recently reported that Hoxa11^+^ lineage cells are ectopic bone-forming MPCs in the burn/tenotomy (BT) traumatic HO model.^24^ Given that the pharmacological inhibition of HIF-1α significantly alleviated traumatic HO,^9^ we first examined the impact of the zeugopod-specific deletion of *Hif1a* on HO formation and the underlying mechanisms. We injected tamoxifen into *Hoxa11-CreER*^T2^ (+); *Hif1a*^*fl/fl*^ mice and littermate control *Hoxa11-CreER*^T2^ (−); *Hif1a*^*fl/fl*^ mice to knockout *Hif1a* in the Hoxa11^+^ lineage cells at 6 weeks of age. At 1-week post-*Hif1a* inactivation, the mice were subjected to a BT injury. At 9 weeks after BT injury, we examined HO deposits at the tenotomy injured limb by µCT imaging (Fig. 1a, b). We found that Hoxa11^+^ cell-specific HIF-1α deletion in adulthood dramatically and significantly decreased HO formation (Fig. 1c). These in vivo data suggest the significance of MPC-specific HIF-1α signaling in ectopic bone formation.

### HIF-1α regulates collagen ECM organization of HO progenitor cells to stimulate HO

**Fig. 2:**
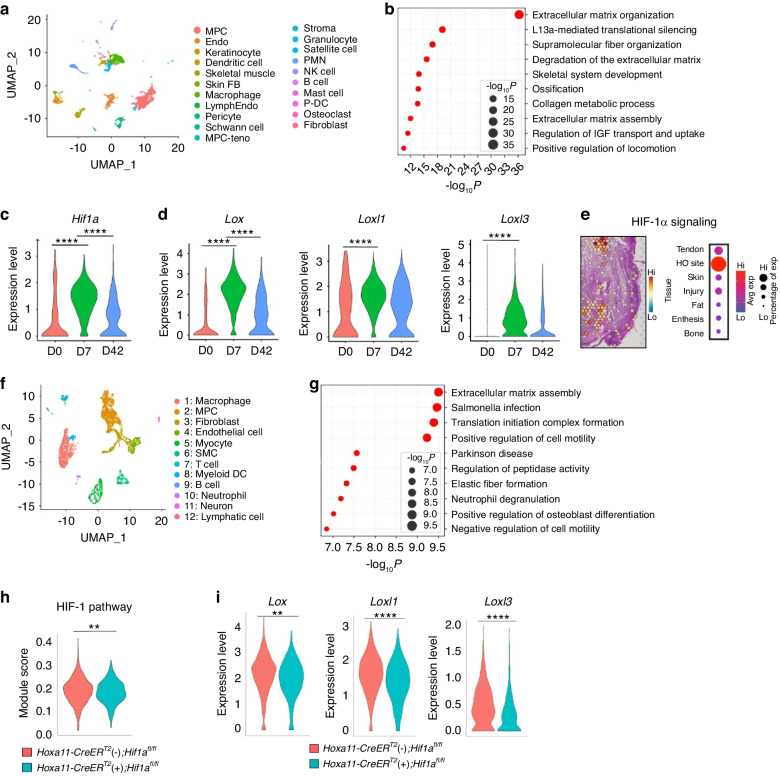
HIF-1α regulates collagen ECM organization to stimulate trauma-induced HO. **a** An scRNA-Seq analysis and integrated UMAP cluster and cluster definition at day 0, 7, and 42 post-BT of *Hoxa11-CreER*^*T2*^*; ROSA-LSL-TdTomato* mice. **b** Gene ontology of upregulated DEGs was analyzed using web-based Metascape. **c** Violin plot of relative expression of *Hif1a* across the time points (D0, D7, and D42) post-BT injury of *Hoxa11-CreER*^*T2*^*; ROSA-LSL-TdTomato* mice. D0 vs. D7, adjusted *P* value = 0; D0 vs. D42, adjusted *P* value = 4.62E-33. **d** Violin plots of relative expression of *Lox, Loxl1*, and *Loxl3* across the time points (D0, D7, and D42) post-BT injury of *Hoxa11-CreER*^*T2*^*; ROSA-LSL-TdTomato* mice. *Lox* (D0 vs. D7, adjusted *P* value = 0; D0 vs. D42, adjusted *P* value = 1.06E-42); *Loxl1* (D0 vs. D7, adjusted *P* value = 1.79E-56); *Loxl3* (D0 vs. D7, adjusted *P* value = 1.38E-123). **e** SpatialFeaturePlot (left) and dot plot (right) showing expression of genes involved in HIF-1α signaling. **f** An scRNA-Seq analysis and integrated UMAP cluster and cluster definition at day 7 post-BT of *Hoxa11-CreER*^*T2*^ (+)*; Hif1a*^*fl/fl*^ and littermate control *Hoxa11-CreER*^*T2*^ *(-); Hif1a*^*fl/fl*^ mice. **g** Gene ontology of DEGs downregulated in *Hoxa11-CreER*^*T2*^ (+)*; Hif1a*^*fl/fl*^ mice 7 days post-BT injury. **h** Modular scoring for the HIF-1 pathway in MPC cluster from *Hoxa11-CreER*^*T2*^ (−)*; Hif1a*^*fl/fl*^ mice and *Hoxa11-CreER*^*T2*^ (+)*; Hif1a*^*fl/fl*^ mice 7 days post-BT. *t* test **P = 0.004. **i** Violin plots of relative expression of collagen cross-linking enzymes in *Hoxa11-CreER*^*T2*^ (−)*; Hif1a*^*fl/fl*^ and *Hoxa11-CreER*^*T2*^ (+)*; Hif1a*^*fl/fl*^ mice 7 days post-BT. *Lox* (adjusted *P* value = 0.002); *Loxl1* (adjusted *P* value = 2.67E-11); *Loxl3* (adjusted *P* value = 1.31E-25)

To determine how zeugopod-specific HIF-1α deletion mitigated HO formation, we subjected cells from the tenotomy injury site of the *Hoxa11*-*CreER*^T2^; *ROSA-LSL-TdTomato* mice to scRNA-seq (Fig. 2a). A comparison of transcript profiles of MPCs prior-to-injury and 7 days-post-BT injury identified a total of 744 differentially expressed genes (DEGs) with 427 upregulated DEGs and 316 downregulated DEGs (log_2_ fold change >0.25; adjusted *P* value < 0.01). Gene ontology (GO) analysis revealed that the up-regulated DEGs were highly enriched in collagen ECM organization, metabolism, motility, and ossification (Fig. 2b). *Hif1a* was among the top 50 highly up-regulated genes. Our time course study using scRNA-seq showed that *Hif1a* expression was highly induced in the MPC cluster in 7 days after injury and declining at the later time point (Fig. 2c and Fig. S1).^9^ Additionally, we analyzed our snATAC-seq dataset and found an increase in open chromatin regions around the promoter region of the *Hif1a* gene consistent with our transcriptomic findings (Fig. S2). The profound effects of hypoxia on ECM homeostasis are displayed in various pathological conditions, such as diabetes and tumorigenesis.^25,26^ Collagen lysyl hydroxylase 2 (PLOD2), an essential collagen-modifying enzyme^12,27,28^ that affects cross-linking by LOX family enzymes of type I and III collagens (the primary collagens at HO sites),^29,30^ is induced upon exposure to hypoxia in a HIF-1α, not HIF-2α, dependent manner. Significant induction of *Plod2* was also seen in our tenotomy injury model (Figs. S2b and S3d).^31,32^ While scRNA-seq provides valuable information concerning cell signaling, it fails to provide spatial information. This spatial context is especially important in musculoskeletal injuries with substantial variability across anatomic/histologic regions. Thus, we turned to spatial transcriptomics using the Visium platform to analyze the tenotomy injury HO site (Fig. 2e and Fig. S4). We segmented the spatial spots on the H&E-stained histological slide (Fig. S4a) into their corresponding tissue types using morphological landmarks (Fig. S4b). Then, to analyze the spatial expression patterns of genes involved in HIF-1α signaling (Fig. 2e and Table S1) and ECM receptors (Fig. S4c and Table S1), we calculated the average expression of these genes of interest at each spatial spot. We observed that HIF-1α signaling genes, and to a lesser extent ECM receptors, were enriched at the HO site with less signaling in uninjured regions of tendon and bone.

Our group and others have previously shown elevated *Hif1a* expression in MPCs during traumatic and genetic forms of HO formation.^9,33^ However, the detailed mechanisms underlying the HIF-1α-mediated regulation of HO progression has yet to be examined. To further elucidate the role of HIF-1α on MPC fate in vivo, we next performed scRNA-seq analysis of an HO-inducing BT in *Hoxa11-CreER*^T2^ (+); *Hif1a*^fl/fl^ mice and control mice (*Hoxa11-CreER*^T2^ (-); *Hif1a*^*fl/fl*^). All cells were isolated from the tenotomy injury site and were subjected to scRNA-seq (Fig. 2f). GO analysis of the 338 DEGs between *Hoxa11-CreER*^T2^ (+); *Hif1a*^*fl/fl*^ and *Hoxa11-CreER*^T2^ (-); *Hif1a*^*fl/fl*^ (log_2_ fold-change > 0.25; adjusted *P* value < 0.01) showed that MPCs from *Hoxa11-CreER*^T2^ (+); *Hif1a*^*fl/fl*^ mice downregulated genes involved in ECM assembly and ossification (Fig. 2g). We also found that *Hif1a* deletion in the Hoxa11^+^ lineage progenitor cells down-regulated gene expression for collagen fibril assembly as well as the HIF-1α pathway (Fig. 2h, i). These results suggest that HIF-1α regulates collagen ECM organization to play a critical role in ectopic bone formation.

### HO-inducing injury increases collagen cross-linking enzyme expression via HIF-1α activation

**Fig. 3:**
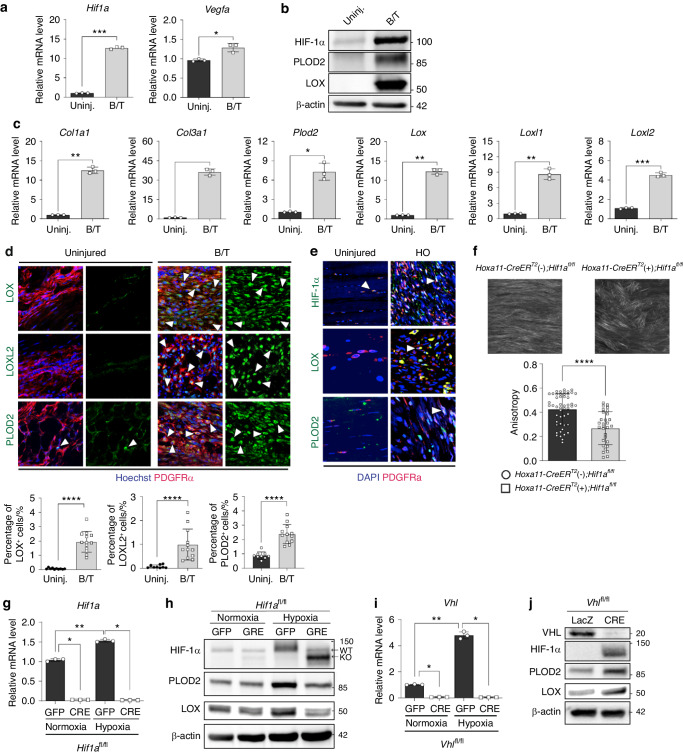
HO-inducing injury increases collagen cross-linking enzyme expression via HIF-1α activation. **a** Real-time qPCR analysis of the expression for *Hif1a* and *Vegfa* at the injury site 7 days post-BT of wild-type mice. *P < 0.05; ***P < 0.000 1. Unpaired *t* test, two-tailed (*n* = 3). **b** Western blot analysis of HIF-1α protein and collagen cross-linking enzymes (PLOD2 and LOX) at the injury site 7 days post-BT of wild-type mice. **c** Real-time qPCR analysis of expression for collagens and collagen cross-linking enzymes at the injury site 7 days post-BT of wild-type mice. *P < 0.05; **P < 0.01; ***P < 0.001. Unpaired *t*-test, two-tailed (*n* = 3). **d** Representative images of immunofluorescent staining of uninjured and injured limb tissue sections for collagen cross-linking enzymes (LOX, LOXL2, and PLOD2). ****P < 0.000 1. Mann-Whitney unpaired *t*-test, two-tailed (*n* = 10–13). **e** Representative images of immunofluorescent staining of human HO tissue and uninjured human tissue for HIF-1α, PLOD2, and LOX. **f** Second harmonic generation imaging of fibrillar collagen at the injury site 1 week post-BT. ****P < 0.000 1. Mann-Whitney unpaired t-test, two-tailed (*n* = 27-30). **g** Real-time qPCR analysis of *Hif1a* expression in MPCs in normoxic or hypoxic conditions. *P < 0.05; **P < 0.01. Mann-Whitney unpaired *t*-test, two-tailed (*n* = 3). **h** Western blot analysis of collagen cross-linking enzyme expression (PLOD2 and LOX) in *Hif1a* KO MPCs in normoxic or hypoxic conditions. **i** Real-time qPCR analysis of *Vhl* expression in MPCs in normoxic or hypoxic conditions. *P < 0.05; **P < 0.01. Mann-Whitney unpaired *t* test, two-tailed (*n* = 3). **j** Western blot analysis of collagen cross-linking enzyme expression (PLOD2 and LOX) in *Vhl* KO MPCs

To further elucidate the underlying mechanism of HIF-1α-regulated HO formation, alterations in gene expression were monitored by real-time qPCR and western blot analyses at the HO site. HIF-1α signaling activation was indicated by elevated expression of *Vegfa*, a HIF-1α target gene, in injured site tissue compared to contralateral uninjured control tissue (Fig. 3a). HO-inducing BT injury led to a significant upregulation of collagens (type I and III) and collagen cross-linking enzymes (PLOD2 and LOX family enzymes; Fig. 3b, c). Immunofluorescent staining of injury site tissue also showed an increased expression of enzymes necessary for collagen cross-linking enzymes (Fig. 3d). To ensure these findings translated to human HO, we next examined samples from individuals with trauma-induced HO compared to non-HO controls and noted a similar increase in HIF-1α and collagen cross-linking enzymes (PLOD2 and LOX; Fig. 3e). To see if this alteration in expression of collagen cross-linking enzymes resulted in a collagen phenotype, we next performed second harmonic generation imaging of the HO site in *Hoxa11-CreER*^T2^(+); *Hif1a*^*fl/fl*^ and control mice (*Hoxa11-CreER*^T2^(−); *Hif1a*^*fl/fl*^). We found that *Hoxa11-CreER*^T2^ (+); *Hif1a*^*fl/fl*^ mice had significantly lower collagen fiber anisotropy compared to control mice (Fig. 3f). This decrease in collagen fiber anisotropy has previously been shown to alter MPC fate away from osteochondral differentiation.^18^ These results suggest that HIF-1α plays a role in regulating collagen cross-linking and ECM organization, thereby affecting MPC fate and HO formation.

Injured Achilles tendon-derived MPCs are the putative osteochondral progenitor cells responsible for HO following a traumatic BT injury. To further elucidate the role of HIF-1α in HO progenitor cells, MPCs isolated from BT-injured *Hif1a*^*fl/fl*^ mice were infected with adenoviral vector-expressing CRE recombinase (Ad-CRE) to knockout *Hif1a* ex vivo and cultured in normoxic and hypoxic conditions (Fig. 3g). *Hif1a* KO MPCs showed diminished expression of collagen cross-linking enzymes (PLOD2 and LOX family). The reduced expression of collagen cross-linking enzymes was even more prominent in hypoxic conditions (Fig. 3h). To further validate the HIF-1α specificity of the results, we also used Von-Hippel Lindau (*Vhl)* KO MPCs to examine the impact of HIF-1α stabilization. VHL is a tumor suppressor and the substrate recognition component of an E3 ubiquitin ligase that targets HIF-1α for polyubiquitination and rapid proteasomal degradation.^34,35^ *Vhl* KO by Ad-CRE infection stabilized HIF-1α and increased gene expression for glycolysis and collagen cross-linking enzymes (Fig. 3i, j). *Vhl* KO MPCs displayed stabilized HIF-1α protein even in a normoxic culture condition (Fig. 3j). These results suggest that HIF-1α activates PLOD2/LOX pathway to regulate collagen ECM organization for HO formation.

### Immobilization suppresses the HO by inhibiting HIF-1α regulation of collagen ECM organization

**Fig. 4:**
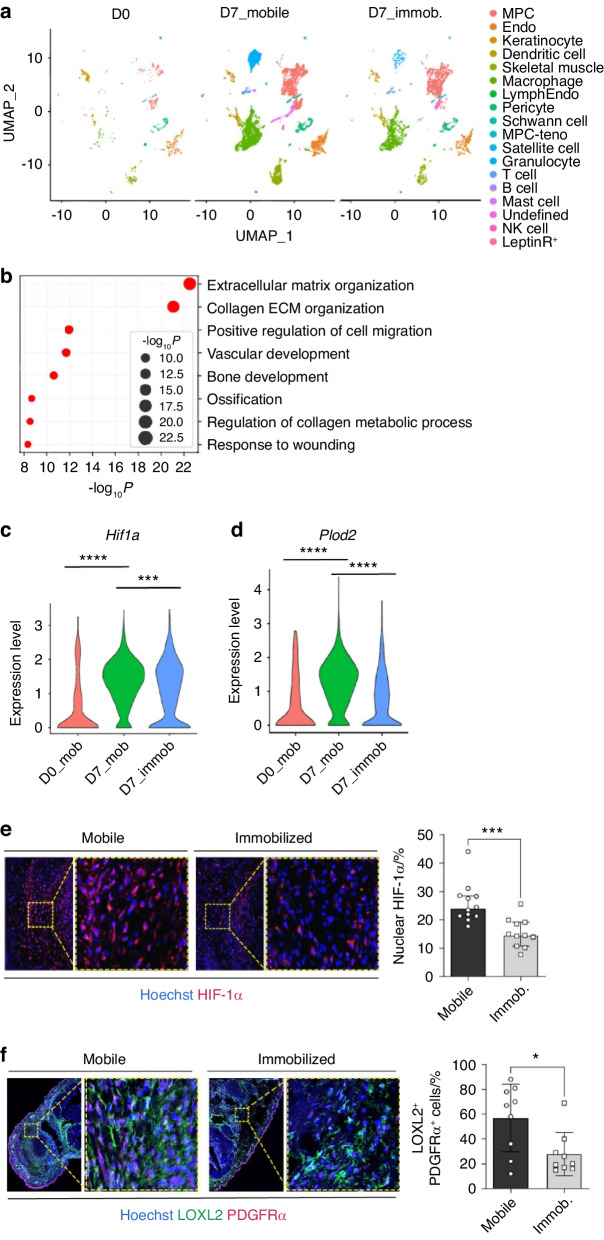
Immobilization suppresses the HO by inhibiting HIF-1α regulation of collagen ECM organization. **a** An scRNA-Seq analysis and UMAP cluster and cluster definitions at day 0 and 7 post-BT injuries with or without limb-immobilization of wild-type mice. **b** Gene ontology of downregulated DEGs was analyzed using Metascape. **c** Violin plot of relative expression of *Hif1a* in MPCs of mobile or immobilized mice 7 days post-BT. D0_Mob vs. D7_Mob, adjusted *P* value = 1.75E-227; D7_Mob vs. D7_Immob, adjusted *P* value = 1.69E-12. **d** Violin plot of relative expression of Plod2 in MPCs of mobile or immobilized mice 7 days post-BT. D0_Mobile vs. D7_Mobile, adjusted *P*-value = 2.65E-24; D7_Mobile vs. D7_Immobilized, adjusted *P* value = 1.77E-91. **e** Representative images of immunofluorescent staining of injured limb tissue sections for HIF-1α protein expression in MPCs. The graph shows the quantification of the HIF-1α signal in MPCs using ImageJ. ***P < 0.000 1. Mann-Whitney unpaired *t* test, two-tailed (*n* = 10). **f** Representative images of immunofluorescent staining of injured limb tissue sections for LOXL2. The graph shows the quantification of the LOXL2^+^ signal using ImageJ. *P < 0.05. Mann-Whitney unpaired *t*-test, two-tailed (*n* = 9)

Given the impact of HIF-1α on collagen ECM modifying enzymes and collagen alignment, we next investigated if this was related to the ECM changes we previously identified from tenotomy site immobilization after BT injury.^18^ Specifically, we found that tendon injury site immobilization decreased collagen fiber anisotropy, which phenocopied our observation made with *Hoxa11-CreER*^T2^ (+); *Hif1a*^*fl/fl*^ mice (Fig. 3f). To examine whether the HIF-1α pathway is implicated in this immobilization-induced process, scRNA-seq data acquired from BT immobilized and mobile control mice were analyzed for HIF-1α pathway and collagen biosynthesis processes (Fig. 4a). Biological pathway enrichment analyses of scRNA-seq datasets identified collagen ECM organization as the highest ranked pathway in the DEGs down-regulated by immobilization (Fig. 4b). *Hif1a* expression was found to be slightly decreased at the transcript level (Fig. 4c) and HIF-1α protein level was significantly decreased at the injury site of immobilized mice (Fig. 4e). The reduced HIF-1α expression was accompanied by significantly reduced expression of *Plod2* (Fig. 4d).^10^ Collagen cross-linking that are derived from hydroxylated lysine residues has increased stability compared with non-hydroxylated lysine residues.^36^ Collagen cross-linking enzyme LOXL2 expression was also decreased in MPCs of immobilized than those of mobilized control mice (Fig. 4f). These results suggest that HIF-1α is involved in the immobilization-mediated suppression of HO by regulating collagen ECM organization for HO development.

### HIF-1α KO most highly impacts glucose metabolism in HO progenitor cells

**Fig. 5:**
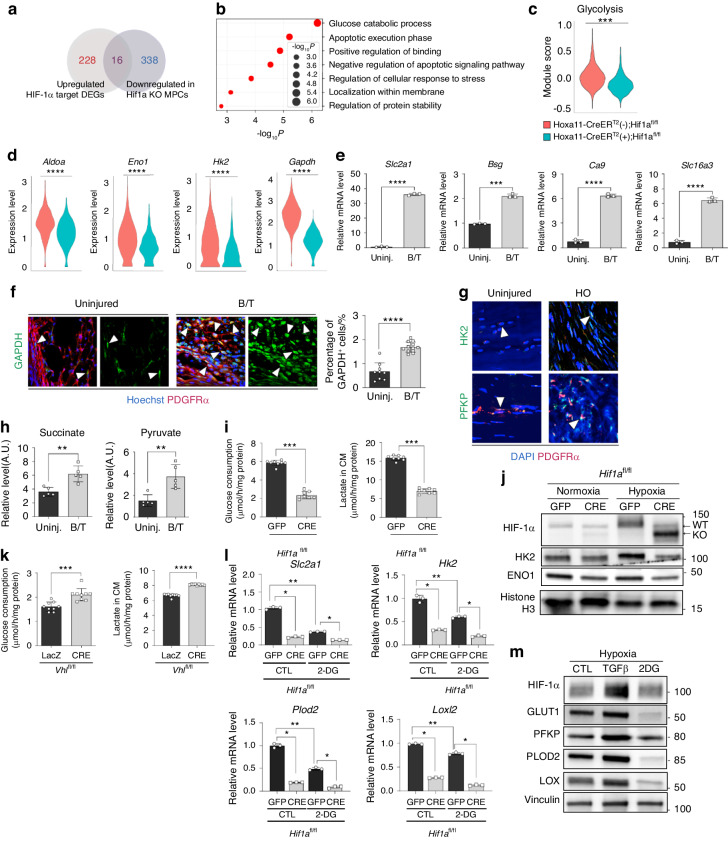
Glucose metabolism is most highly impacted by HIF-1α KO in HO-forming MPCs. **a** Venn diagram of HIF-1α target genes among upregulated DEGs in *Hoxa11-CreER*^*T2*^*; ROSA-LSL-TdTomato* by BT injury *vs*. downregulated DEGs in *Hoxa11-CreER*^*T2*^(+)*; Hif1a*^*fl/fl*^. **b** Gene ontology of 16 DEGs from (a) by Metascape. **c** Modular scoring for glycolysis pathway in *Hoxa11-CreER*^*T2*^(-)*; Hif1a*^*fl/fl*^ *mice* and *Hoxa11-CreER*^*T2*^(+)*; Hif1a*^*fl/fl*^ mice 7 days post-BT. *t*-test, *P* value < 2.2e-16. **d** Violin plots of glycolysis enzymes in *Hoxa11-CreER*^*T2*^ (-); *Hif1a*^*fl/fl*^ mice and *Hoxa11-CreER*^*T2*^(+)*; Hif1a*^*fl/fl*^ mice 7 days post-BT. *Aldoa* (adjusted *P* value = 1.34E-47); *Eno1* (adjusted *P* value = 3.85E-21); *Gapdh* (adjusted *P*-value = 1.75E-227); *Hk2* (adjusted *P* value = 3.63E-33). **e** Real-time qPCR analysis of expression for glycolysis-associated genes at the injury site of a wild-type mouse 7 days post-BT. ***P < 0.001, ****P < 0.000 1. Unpaired *t*-test, two-tailed (*n* = 3). **f** Representative images of immunofluorescent staining of injured limb tissue sections for glycolysis enzyme GAPDH. ****P < 0.000 1. Mann-Whitney unpaired *t*-test, two-tailed (n = 10-13). **g** Representative images of immunofluorescent staining of human HO tissue and uninjured human tissue for glycolysis enzymes (HK2 and PFKP). **h** Untargeted metabolomics analysis of HO site tissue 7 days post-BT of wild-type mice. Graphs show levels of pyruvate and succinate at the injury site 7 days post-BT. **P < 0.01. Mann-Whitney unpaired *t*-test, two-tailed (*n* = 5). **i** Glucose consumption and lactate production by *Hif1a* KO and WT MPCs in hypoxic conditions. *Hif1a* KO MPCs were generated by adenoviral expression of CRE recombinase (Ad-CRE) in *Hif1a*^*fl/fl*^ MPCs. ***P < 0.001. Mann-Whitney unpaired *t* test, two-tailed (*n* = 8). **j** Western blot analysis of glycolysis enzyme expression (HK2 and ENO1) in *Hif1a* KO MPCs. Histone H3 served as an internal control. **k** Glucose consumption and lactate production by *Vhl* KO and WT MPCs. ***P < 0.001; ****P < 0.000 1. Mann-Whitney unpaired *t* test, two-tailed (*n* = 9). **l** Real-time qPCR analysis of expression for glycolysis (GLUT1 and HK2) and collagen cross-linking enzymes (PLOD2 and LOXL2) in MPCs. *P < 0.05; **P < 0.01. Mann-Whitney unpaired *t*-test, two-tailed (*n* = 3). **m** Western blot analysis of expression of collagen cross-linking enzymes (PLOD2 and LOX) after treating wild-type MPCs with 2-DG to block glycolysis

Given that energy is necessary to support collagen deposition and collagen cross-linking,^37^ we next queried the impact of HIF-1α on cellular metabolism. In hypoxia, HIF-1 activation inhibits the mitochondrial pyruvate dehydrogenase (PDH) to repress mitochondrial oxidative phosphorylation and the pyruvate from glycolysis is converted into lactate, which is exported to the extracellular space.^38,39^ Among the 823 upregulated DEGs in 7 days post-injury of wild-type (WT) mice, 228 DEGs were known to be target genes of HIF-1α (log_2_ fold change > 0.25; adjusted *P* value < 0.01). Sixteen of the 228 DEGs were further found to be suppressed by *HIf1a* deletion in *Hoxa11-CreER*^T2^ (+); *Hif1a*^*fl/fl*^ (Fig. 5a). GO analysis of the 16 DEGs revealed that glucose metabolism was the most impacted pathway by HIF-1α depletion (Fig. 5b). Module score analysis indicated that glycolysis pathway gene expression was decreased in MPCs of *Hoxa11-CreER*^T2^ (+); *Hif1a*^*fl/fl*^ MPCs (Fig. 5c, d). Increased expression of *Slc2a1* (*a.k.a*. glucose transporter 1 [GLUT1]), Basigin (*Bsg, a.k.a*. CD147), carbonic anhydrase 9 (*Ca9*), and *Slc16a1* (*a.k.a*. monocarboxylic acid transporter 4 [MCT4]) suggested elevated glucose uptake, glycolysis, and lactate efflux at the tenotomy injury site (Fig. 5e).^40^ Consistently, immunofluorescent staining showed that injured tissues expressed higher levels of glycolytic enzyme, GAPDH, compared to uninjured tissues (Fig. 5f). Human HO tissue expressed increased levels of glycolytic enzymes compared to uninjured control tissue (Fig. 5g). Metabolomics analysis of tenotomy site tissue revealed increased pyruvate levels in the injury site compared to that of uninjured controls, indicating metabolic reprogramming with increased glycolysis (Fig. 5h). The metabolite succinate was also increased at the injury site (Fig. 5h). Succinate is produced during oxidation reactions by α-ketoglutarate (αKG)-dependent dioxygenases like PLOD2 and is known to stabilize HIF-1α.^41^ In vitro cultures of *Hif1a* KO MPCs showed significantly decreased glucose uptake and lactate production (Fig. 5i) and decreased expression of key enzymes involved in the glycolysis pathway (HK2 and ENO1) from the cells cultured in hypoxic conditions (Fig. 5j). Conversely, *Vhl* KO MPCs showed increased glucose consumption and lactate production (Fig. 5k). These results suggest that metabolic reprogramming by HIF-1α activation towards glycolysis plays a role in HO progenitors to promote traumatic HO.

To explore whether glycolytic metabolism and collagen ECM organization are mechanistically coupled in MPCs for HO progression, we further examined the expression of glycolysis and collagen cross-linking enzymes after the treatment with 2-deoxy-D-glucose (2-DG). 2-DG is a non-metabolizable glucose analog that interferes with glycolysis metabolism. The decreased expression for glycolysis and collagen cross-linking enzymes in *Hif1a* KO MPCs was further alleviated by 2-DG treatment (Fig. 5l). To further assess the effect of metabolic reprogramming on collagen fibril assembly, MPCs were treated with 2-DG in hypoxic conditions. The blockage of glycolysis significantly suppressed the expression of collagen cross-linking enzymes, while TGF-β1, a well-known stimulator of collagen production, significantly increased collagen cross-linking enzyme expression (Fig. 5m). Glycolytic enzymes and HIF-1α protein levels were also increased in hypoxic conditions, suggesting the potential cooperative effects of TGF-β signaling and HIF-1α activation in HO. These results indicate that HIF-1α connects glycolytic metabolism and collagen biosynthesis in MPCs for HO formation and progression.

### The HIF-1α/PLOD2 pathway regulates the osteogenic differentiation of HO progenitor cells

**Fig. 6:**
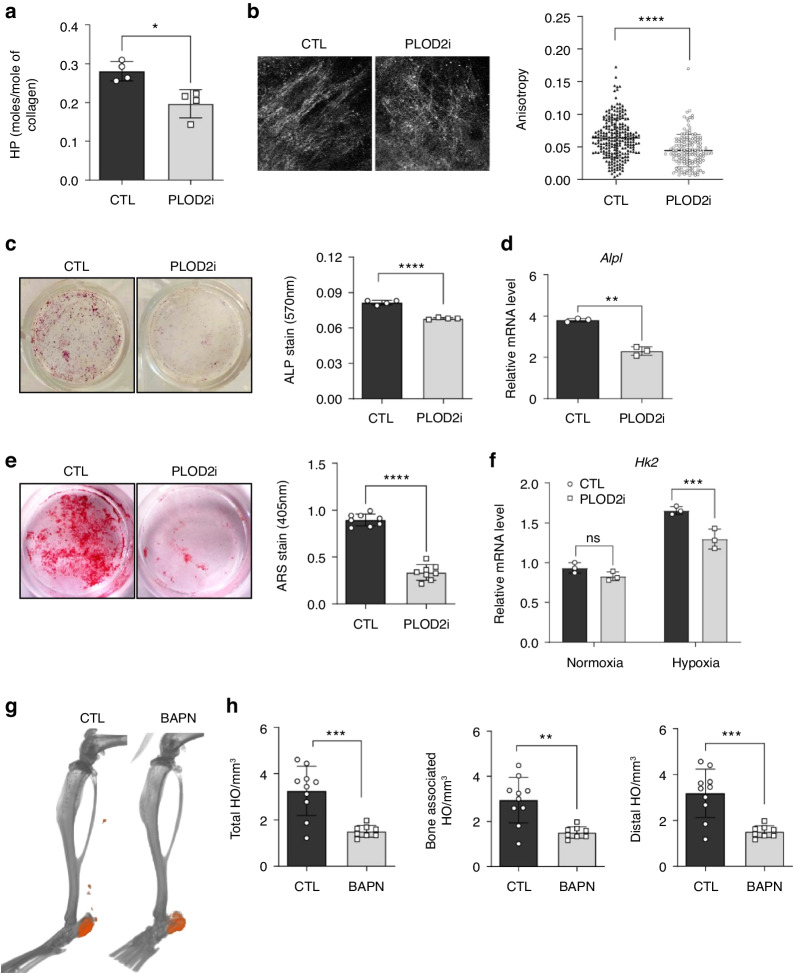
The HIF-1α/PLOD2 pathway regulates cell motility and osteogenic differentiation of HO progenitor cells ex vivo. **a** The pyridinoline cross-link content was determined by HPLC of cell-matrix harvested 4-weeks treatment of cells with ascorbate-2-phosphate in the presence of PLOD2i. Pyridinoline cross-linking was significantly decreased in the cell/matrix of MPCs treated with PLOD2i for 4 weeks. The concentration of hydroxylysine pyridinoline (HP) cross-linking residues is expressed as moles/mole of collagen. *P < 0.05. Mann-Whitney unpaired *t* test, two-tailed (*n* = 3). **b** Collagen deposited by cultured MPCs was immunostained with a collagen α1[I] C-telopeptide antibody. The collagen matrix in MPCs treated with PLOD2i appears disorganized, while vehicle (DMSO) control appears to have aligned fibrillar collagen strands (*n* = 2, representative panels shown). The graph shows the quantification of anisotropy. Error bars represent mean ± SD. ****P < 0.000 1. Mann-Whitney unpaired *t*-test, two-tailed (*n* = 20). **c** Representative images of alkaline phosphatase (ALP) staining. MPCs were subjected to osteogenic differentiation for 7 days in the presence of PLOD2i or vehicle (DMSO). The graph quantifies ALP-staining intensity by measuring colorimetric absorbance at 570 nm wavelength. The results are expressed as the mean ± SD. ****P < 0.000 1. Mann-Whitney unpaired *t*-test, two-tailed (*n* = 4). **d** Real-time qPCR analysis of *Alpl* expression. MPCs isolated from a wild-type BT mouse were subjected to osteogenic differentiation for 7 days in the presence of a PLOD2 inhibitor or vehicle (DMSO). Error bars represent mean ± SD. **P < 0.01, versus vehicle control. Mann-Whitney unpaired *t*-test, two-tailed (*n* = 3). **e** Representative images of in vitro mineralization of MPCs. MPCs were subjected to osteogenic differentiation in the presence of PLOD2i or vehicle (DMSO). After 6 weeks in culture, mineralization was visualized by Alizarin Red S (ARS) staining. ARS stain was quantified by measuring colorimetric absorbance at 407 nm wavelength. The results are expressed as the mean ± SD. ****P < 0.000 1. Mann-Whitney unpaired *t*-test, two-tailed (*n* = 8). **f** Real-time qPCR analysis of Hk2 expression. MPCs isolated from a wild-type BT mouse were cultured in normoxic or hypoxic conditions in the presence of a PLOD2 inhibitor. Error bars represent mean ± SD. ***P < 0.001, versus vehicle control. Mann-Whitney unpaired *t*-test, two-tailed (*n* = 3). **g** Representative 3-D reconstruction μCT images of injured limbs from BAPN-treated mice and vehicle (5% sucrose)-treated mice 9 weeks post-BT. Ectopic bone formation was indicated by pseudo-coloring (orange) the bone. **h** Quantification of total, bone-associated, and distal HO. Error bars represent mean ± SD. **P < 0.01; ***P < 0.001. Mann-Whitney unpaired *t*-test, two-tailed (*n* = 10/group)

Based on the findings from our scRNA-seq, in vivo, and ex vivo studies, we hypothesized that collagen cross-linking by the PLOD2-LOX pathway could be one of the modalities by which HIF-1α stimulates osteochondral differentiation of MPCs in traumatic HO. A recent high-throughput small-molecule drug screen identified compounds using a novel luminescence-based assay that measures PLOD2 activity based on the amount of succinate produced from α-KG, which is required for enzymatic activities for oxygenase family protein like PLOD2.^42^ The PLOD2 inhibitor was tested in a recently reported study, showing that the inhibition of PLOD2 activity decreased cell proliferation in lung cancer cells ex vivo.^43^ To further test our hypothesis on the vital role of the HIF-1α/PLOD2 axis in traumatic HO, we utilized the PLOD2 inhibitor to characterize the biological implications of perturbed collagen alignment on HO-forming MPCs. We first evaluated the effect of this PLOD2 inhibitor on collagen fiber alignment. Primary cultures of MPCs from tenotomy injury sites were treated with the PLOD2 inhibitor, and collagen hydroxylysine pyridinoline (HP) contents were determined by high-performance liquid chromatography (HPLC). The MPCs treated with the PLOD2 inhibitor deposited ECM with less collagen HP compared to vehicle controls (Fig. 6a). Consistently, immunostaining of MPC-derived ECM for type I collagen showed disorganized collagen fibers in PLOD2 inhibitor-treated cultures compared to vehicle control cells (Fig. 6b). In addition, WT MPCs cultured in hypoxic condition and *Vhl* KO MPCs displayed more aligned type I collagen fibers compared to WT MPCs in normoxic condition (Fig. S5). Thus, the impact of PLOD2 inhibition on ECM alignment phenocopies the results from *Hif1a* deletion (Fig. 3f) and joint immobilization.^18^ Given the effects of PLOD2 inhibition on collagen alignment, we next evaluated the effect of PLOD2 inhibition on the osteogenic potential of HO-forming MPCs. MPCs were cultured in osteogenic media in the presence of the PLOD2 inhibitor for 7 days. Then, alkaline phosphatase (ALP) gene expression and activity were measured by ALP staining and real-time qPCR, respectively. MPCs treated with the PLOD2 inhibitor showed significantly lower levels of *Alp* expression and ALP activity at 7 days post-induction than vehicle control (Fig. 6c, d). To further test if PLOD2 functional inhibition impairs collagen ECM mineralization during osteogenesis, MPCs from wild-type BT injury mice were cultured in osteogenic conditions. We found that PLOD2 inhibitor-treated cultures mineralized poorly until day 28 of differentiation and that the amount of mineralized matrix, assessed by alizarin red staining, was largely decreased compared with vehicle control by 42 days of osteogenic differentiation (Fig. 6e). These results are in line with the recent report that *Plod2*‑null MC3T3 cells displayed diminished collagen stability with reduced fibril diameter and defective mineralization.^44^

Given our above studies with cultured MPCs revealing that HIF-1α activation regulates glycolysis and collagen cross-linking, we next evaluated the impact of PLOD2 inhibition on glycolytic metabolism. MPCs were treated with the PLOD2 inhibitor, and the expression of glycolytic enzymes was examined by real-time qPCR. The expression of *Hk2*, which encodes hexokinase 2 for catalyzing the rate-limiting first step of glycolysis, was significantly decreased by PLOD2 inhibition (Fig. 6f), suggesting that PLOD2 regulates glucose metabolism via controlling HK2 expression. In agreement with our finding, PLOD2 was shown to upregulate HK2 expression, thereby affecting the proliferation, migration, and invasion of colorectal cancer cells.^45^ These results suggest that the HIF-1α/PLOD2 axis couples glucose metabolism and collagen matrix organization in a hypoxic microenvironment during HO progression.

### Inhibition of collagen cross-linking enzymes suppresses BT-induced HO in vivo

Collagen cross-linking is implicated in cell communication with the ECM through mechano-transduction and thereby plays a critical role in MPC fate determination.^46^ To assess the impact of collagen cross-linking interruption on HO formation, 7-week-old male C57BL/6 J mice were subjected to BT injury followed by β-aminopropionitrile (BAPN), an irreversible inhibitor of LOX, administration via drinking water ad libitum. BAPN downregulated the expression of PLOD2 and inhibited the formation of a stable collagen matrix by directly inhibiting LOX family members.^47^ HO on the injured limb was measured at 9 weeks post-injury. BAPN-administered mice showed significantly reduced levels of HO, including calcaneus bone-associated and tendon-associated HO compared to the control mice (Fig. 6g, h). Our findings have identified the HIF-1α/PLOD2/LOX axis as a promising HO therapeutic target important in integrating glucose metabolism and collagen ECM organization.

## Discussion

HO is abnormal bone formation within non-osseous skeletal tissues and is known to be initiated by inflammation upon traumatic tissue injuries. Although various cellular and molecular pathways have been known to contribute to this ectopic bone formation, the pathophysiological mechanisms of traumatic HO are still unclear. We and others previously reported that HIF-1α is a potent stimulus of HO.^9,33,48^ However, an incomplete understanding of the molecular mechanisms underlying HIF-1α regulation of HO progression limits therapeutic advances. In the present study, we utilized single-cell and spatial transcriptional profiling and our well-established BT mouse model to identify the HIF-1α/PLOD2 axis in MPCs as a critical driver of traumatic HO. Mechanistically, HIF-1α activation directly induced the expression of PLOD2 and the downstream collagen cross-linking enzymes (LOX family) to affect the alignment of the collagen fibrils. We also showed that the HIF-1α/PLOD2 pathway is associated with the reciprocal crosstalk between glycolytic metabolism and collagen ECM organization in HO-forming MPCs (Fig. S6). Based on our in vivo and ex vivo findings in the present study, we propose collagen cross-linking enzymes as potential therapeutic targets to treat traumatic HO.

HO is a multifactorial process involving the interplay among contributing cell types including HO-forming MPCs.^1^ The hypoxic microenvironment and resulting HIF-1α activation provide a permissive microenvironment for HO-forming MPCs to undergo osteochondral differentiation, ultimately leading to ectopic bone formation.^9^ Inflammation and the subsequent occurrence of local tissue hypoxia are the early events of traumatic HO.^49^ HIF-1α was shown to play a critical role specifically at the early stage of HO and co-inhibition of HIF-1α and RUNX2 was more effective than HIF-1α, inhibition alone in preventing HO formation.^50^ The significance of HIF-1α activation in HO has also been shown in fibrodysplasia ossificans progressiva (FOP), a genetic cause of HO^51^ where the HIF-1α pathway amplifies BMP signaling and acts as an integral point of inflammation and local tissue hypoxia in HO of FOP.^33^ Therefore, targeting the HIF-1α pathway may offer the most efficient strategies to prevent HO.

Our bioinformatics analysis of scRNA-seq and spatial transcriptomic data revealed that the HO-stimulating effect of HIF-1α occurs mainly through the regulation of collagen biosynthesis and ECM organization. Collagen ECM regulation in hypoxia requires the stimulation of collagen synthesis and post-translational modification. HIF-1α, among other hypoxia-inducible factors, was described as a critical regulator of collagen ECM composition, alignment, and mechanical properties by promoting the expression of collagen prolyl hydroxylases (P4HA1 and P4HA2), lysyl hydroxylases (PLOD2), and lysyl oxidases (LOX and LOXLs) in human fibroblasts and cancer cells.^10,52^ Consistent with the role of TGF-β1 in HO formation,^53^ HIF-1α activation and TGF-β1 cooperated to increase collagen ECM deposition in lung fibrosis.^54^ More specifically, HIF-1α pathway activation was important for the post-translational modification of collagen and pyridinoline cross-linking altering collagen fiber alignment, while TGF-β1 had a dominant role in increasing the collagen synthesis per se.^54^

HIF-1α activation under hypoxic conditions recruited HO precursor cells and directly impacted MPC fate decisions to induce ectopic bone formation.^48,55,56^ As the major microenvironment component, collagen ECM is involved in cell survival, proliferation, adhesion, migration, and differentiation affecting the fate of HO-forming MPCs. A previous study using a fibroblast cell line demonstrated that HIF-1α stabilization in hypoxia resulted in enlarged cell volume and increased focal contact numbers.^57^ We showed that collagen fibril alignment was altered by both *Hif1a* KO and PLOD2 inhibition.

HIF-1α is the main driver of metabolic adaptation in hypoxia.^58^ HIF-1α-induced gene products reprogram cellular metabolism activating the glycolysis pathway and suppressing oxidative phosphorylation in the mitochondria in hypoxia. The metabolic regulation of ECM homeostasis has been described in several pathological contexts, such as the upregulation of glycolysis and downregulation of fatty acid oxidation increased ECM deposition in skin fibrosis.^59^ The knockdown of pyruvate kinase M2 (PKM2) downregulated the expression of COL2A1 and SOX9 to alter the ECM of chondrocytes in osteoarthritis.^60^ Conversely, ECM stiffness regulates cellular glucose metabolism mediated by multiple pathways, including the YAP/TAZ pathway, integrin/FAK pathway, and ZFP36/TXNIP/GLUT1 signaling.^61,62^ Although the role of HIF-1α in cellular energy metabolism has been extensively studied, whether and how HIF-1α is involved in the reciprocal crosstalk between ECM organization and cell metabolism is underexplored. Our findings from in vivo metabolomics and ex vivo studies suggest the implication of the HIF-1α/PLOD2 pathway in the metabolic regulation of collagen cross-linking in HO-forming MPCs. Consistent with our findings, *Plod2* knockdown decreased glycolytic energy metabolism by suppressing HK2 expression in MPCs, leading to the alteration of MPC migration and chondrogenic potential in vitro.^63^ PLOD2 also promotes aerobic glycolysis and cell progression in colorectal cancer by upregulating HK2.^45^ In addition, elevated PLOD2 expression was associated with increased cytoplasmic succinate levels for cancer cell mesenchymal phenotypes and stemness,^28^ further supporting our finding that the HIF-1α/PLOD2 axis regulates cell metabolism to affect the fate of HO-forming MPCs.

There are some limitations in the present study. First, we observed that stabilized HIF-1α reprogrammed cellular metabolism by activating glycolysis. However, how HIF-1α KO in HO-forming MPCs affects mitochondrial energy metabolism with diminished glycolytic metabolism remains to be determined. Prolonged HIF-1α activation in chondrocytes was shown to alter cellular bioenergetics, thereby metabolically controlling collagen synthesis and modification.^64^ Second, although we clearly demonstrated that the HIF-1α/PLOD2 pathway orchestrates glycolytic metabolism and collagen ECM organization at the gene expression level, it is currently unclear whether collagen-modifying functionality of PLOD2 is required for the regulation of glycolysis or whether there are other unidentified functions exerted by lysyl hydroxylase activity of PLOD2. Lastly, we did not show how pan-LOX inhibitor (BAPN) affected cellular metabolism in vivo and/or ex vivo. LOX promotes tumor cell growth and proliferation by participating in Warburg effect-mediated metabolic signaling.^65^ Recombinant LOX protein treatment increased glucose consumption and lactate production by human gastric cancer cell lines. Whether LOX’s intracellular lysyl oxidase activity plays a role in metabolic regulation remains to be further examined.

In conclusion, this study elucidates the molecular mechanisms by which the hypoxia/HIF-1α/PLOD2/LOX pathway contributes to traumatic HO. Due to the vast network of downstream pathways regulated by HIFs to adapt to hypoxia, more specific approaches targeting the HIF pathways are necessary for effective HO treatment. By searching for alternative targets downstream of HIF-1α activation, this study identified the HIF-1α/PLOD2/LOX axis, a previously undescribed pathway in traumatic HO, as the critical driver of HO progression. We proposed PLOD2/LOX as a target for therapeutic intervention and a candidate biomarker for monitoring traumatic HO occurrence. Furthermore, our study provides insight into the reciprocal crosstalk between ECM and metabolism.

## Materials and methods

### Mice

The University of Texas Southwestern Medical School Institutional Animal Care and Use Committee reviewed and approved all mice studies. *Hoxa11*-*CreER*^T2^ (+) were crossed with *Hif1a*^fl/fl^ mice to yield *Hoxa11*-*CreER*^T2^ (+); *Hif1a*^fl/fl^ mice and *Hoxa11*-*CreER*^T2^ (-); *Hif1a*^fl/fl^ mice. *Hoxa11*-*CreER*^T2^ (-); *Hif1a*^fl/fl^ mice were used as the littermate controls. To induce a CRE recombination-mediated deletion of *Hif1a*, tamoxifen (T5648, Sigma) was dissolved in 100% ethanol (100 mg/mL) and then diluted to working concentration (20 mg/mL) in corn oil (Sigma). Tamoxifen (75 mg/kg body weight) was administered via intraperitoneal daily injection for 5 days starting at 6 weeks of age. After one week of tamoxifen washout, a BT procedure was performed. For the BAPN study, drinking water for mice was prepared by adding BAPN (3-Aminopropionitrile, stabilized, AC351750250, Thermo Scientific Chemicals) to sterilized water containing 5% sucrose (S0389, Sigma).

### BT injury and joint immobilization

As previously described, mice underwent a 30% total body surface area backburn with concurrent Achilles’ transection^66^. Mice were anesthetized with isoflurane and were administered analgesia (1.2 mg/kg buprenorphine SR) via subcutaneous injection before the injury. The uninjured right leg served as an uninjured internal control. Joint immobilization was performed immediately after injury. Briefly, the left hind limb was maintained in a knee joint extension and ankle plantar extension position and taped with a commercially available hook-and-loop fastener (Velcro® One-Wrap straps) around the limb. Mice were single-housed during the study.

### Bioinformatics analysis and visualization of scRNA-Seq and snATAC-Seq data

Three different scRNA-seq datasets, two previously published datasets^18,24^ and one new dataset from *Hoxa11*-*CreER*^T2^ (+); *Hif1a*^*fl/fl*^ mice and *Hoxa11*-*CreER*^T2^ (-); *Hif1a*^*fl/fl*^ mice, were used in this manuscript. Samples, library preparation, and sequencing methods for scRNA-seq and snATAC-seq datasets were previously described^24^. The analysis using scRNA-seq data, shown in Fig. 2 and Fig. 5, was performed on samples from *Hoxa11*-*CreER*^T2^ (+); *Hif1a*^fl/fl^ mice and *Hoxa11*-*CreER*^T2^ (-); *Hif1a*^fl/fl^ mice. Cell Ranger (version 5.0.1) software from 10X Genomics Inc. was used to align reads to mm10-1.2.0 reference genomes to generate matrices for Prox1-eGFP samples. Cells with abnormally high or low expressed genes, cells with excessive UMI counts, cells with more than 20% mitochondrial UMI counts, and cells with less than 5% ribosome UMI counts were removed for each sample. After quality control, we applied Seurat’s (v4.2.0) integration workflow to correct possible batch effects for the remaining cells of all the samples. Genes with high variance were selected using the “FindVariableGenes” function for dimensionality reduction. Cells were clustered with Seurat’s “FindClusters” function. Cell types were identified for each cluster using the cell type maker genes.^24^ Gene expression was normalized for all visualizations. Gene enrichment analysis was performed using the web-based platform Metascape.^67^

### Spatial transcriptomics

Histological sections of the tenotomy injury site were placed onto Visium spatial gene expression system slides (10X Genomics), which contain spatial spots with unique oligonucleotide primers that identify the location where transcripts are captured. Routine H&E staining and imaging was performed, before the histological sections were subjected to probes from a library of over 20 000 mouse genes. Bound probes were hybridized, rinsed, eluted, and sequenced. The resulting sequencing was aligned to the H&E images using the spatial barcodes. Using the morphological features on the H&E-stained slide, spatial spots were segmented to their corresponding tissue type in the Loupe Browser application. Basic spatial transcriptomic pre-processing was performed using the R package Seurat, and expression analysis of various gene lists was performed. Using AddModuleScore^68^ function, scores representing the average expression of genes were calculated for HIF-1α signaling, ECM receptors, and ECM components.

### Metabolomic profiling

Tissue samples were extracted from the tenotomy injury site 7 days post-injury followed by untargeted metabolomics analysis by liquid chromatography and high-resolution mass spectrometry (LC-MS) as described previously.^69^

### Micro CT and quantification of ectopic bone formation

Full hindlimb samples from subject mice were harvested at 9 weeks following BT injury. Legs were fixed in 4% paraformaldehyde (PFA, in PBS) for 18 h at 4 °C, washed with 1 × PBS. μCT images were taken at the University of Texas Southwestern Department of Radiology using a nanoScan PET/CT system (Mediso USA). Images were quantified using Dragonfly (Object Research Systems) as previously described.^19^

### Histology and immunofluorescent staining

Based on published methods, hindlimbs were fixed, processed, and frozen^19^. Ten-micron thick frozen longitudinal hindlimb sections were used for immunofluorescent staining. Immunofluorescent staining was performed using published methods.^19^ Confocal microscope images were captured using a Leica Stellaris DMi8 microscope using the HC PL APO CS2 20× objective and 3.15× digital zoom to create a 63× image for analysis. Raw channel images for Hoechst and antigens of interest were exported using Leica Application Suite software. Each raw image was analyzed as an 8-bit image, and the percent area was quantified using the same manual thresholding parameters within each experiment in ImageJ software.

### Second harmonic generation imaging

Multiphoton microscopic imaging was used to survey collagen at the injury site, as described previously.^18,70^ Cryopreserved 30 μm sections on slides were washed in 0.05% TBS-T to remove OCT from the section. Slides were then mounted with water-based mounting media, covered with glass coverslips, and sealed with nail polish. Slides were imaged using a Leica SPX8 confocal laser scanning microscope. Collagen fibrillar structures were analyzed using the ImageJ plugin, FibrilTool, to quantify array orientation and anisotropy.^71^

### MPC isolation and tissue culture

The tissue at the tenotomy site was dissected and digested with collagenase as described in our previous report.^18^ Adherent cells were expanded by culturing in DMEM supplemented with 20% FBS (Thermo Fisher Scientific). Cells of passages 2 and 3 were used in this study.

### Collagen matrix and HP

Cells and matrix from cultured mouse MPCs were harvested in PBS containing protease inhibitors (Pierce) after 4 weeks in collagen deposit media (DMEM, 10% FBS, and ascorbic-2-phosphate (300 μmol/L). Cells were resuspended in Milli-Q H_2_O and pelleted by centrifugation. The pyridinoline cross-link content was determined by HPLC after acid hydrolysis in 6 mol/L HCl for 24 h at 108 °C. Lyophilized samples were dissolved in 1% (v/v) n-heptafluorobutyric acid and their reverse-phase HPLC hydroxylysine pyridinoline (HP) contents were quantified by fluorescence monitoring as previously described.^72^

### Statistical analysis

Statistical analysis was performed with the GraphPad Prism software (ver.10.0). To determine significance, a two-tailed unpaired student’s *t*-test was performed. The graphs show significance as follows: *P > 0.05, **P > 0.01, ***P > 0.001, ****P > 0.000 1.

### Supplementary information

Supplementary information

## Data Availability

Previously published scRNA-seq datasets,^18,24^ snATAC-seq datasets,^24^ one new scRNA-Seq dataset from *Hoxa11*-*CreER*^T2^; *Hif1a*^fl/fl^ mice, and spatial transcriptomics dataset are available at NCBI GEO (https://www.ncbi.nlm.nih.gov/geo).
