# Supplementary material for: The HIF-1α/PLOD2 axis integrates extracellular matrix organization and cell metabolism leading to aberrant musculoskeletal repair
Source: Bone Res. 2024 Mar 12;12:17. doi: 10.1038/s41413-024-00320-0 (PMC10933265; doi:10.1038/s41413-024-00320-0)

**Supplementary information for:**

**The HIF-1α/PLOD2 axis integrates extracellular matrix organization and cell metabolism leading to aberrant musculoskeletal repair**

Heeseog Kang 1, Amy L. Strong 2, Yuxiao Sun 1, Lei Guo 3, Conan Juan 1, Alec C. Bancroft 1, Ji Hae Choi 1, Aysel A. Fernandes 4, Michael Woodard 1, Juhoon Lee 5, Sowmya Ramesh 6, Aaron W. James 6, David Hudson 4, Kevin N. Dalby 5, Lin Xu 3, Robert J. Tower 1, Benjamin Levi 1, *

* Corresponding author: Benjamin Levi

Email: Benjamin.Levi@UTSouthwestern.edu

This file includes:

Supplementary information text (Materials and methods)

Figures and legends S1 to S6

Table S1

**Supplementary information text**

**Materials and methods**

**Human tissue histology and immunofluorescent staining**

For human tissue immunofluorescence, 5 micron-thick paraffin sections of human tendon HO from a 37-year-old male with HO (healthy tendon tissue was used as an uninjured control) were deparaffinized in xylene, and a series of graded ethanol solutions and rehydrated using water. The slides were treated with Proteinase K (S3020, DAKO) for 12 minutes at RT and then incubated with 10% goat serum (1h at RT). The following antibodies were used: anti-HIF-1α (1:1000, Rabbit monoclonal; 36169, Cell Signaling Technology), anti-PLOD2 (1:1000, rabbit polyclonal; 21214-1-AP, Proteintech), anti-LOX (1:800; rabbit monoclonal; ab174316), anti-HK2 (1:800; rabbit polyclonal; PIPA587552, Thermo Scientific), anti-PFKP (1:50; NBP2-01539, NOVUS biological), anti-PDGFRα (1:1000; ab96569, abcam) and secondary antibodies conjugated with goat anti-rabbit IgG H&L Alexa Fluor® 568 (1:3000; ab175471, abcam) and goat anti-mouse IgG H&L Alexa Flour® 647 (1:3000; ab150115, abcam). Digital images of these immunofluorescent-stained sections were captured with 20× and 40× objectives using upright fluorescent microscopy (Leica DM6) coupled with a camera (DFC3000G). Microscopic digital images of the immunofluorescence-stained sections were captured with 20×, 40x objectives using upright fluorescent microscopy (Leica DM6, camera DFC3000G).

**RNA extraction and real-time qPCR**

Total RNA was extracted from mouse tissues or cultured MPCs using the RNeasy mini kit (Qiagen) following the manufacturer’s instructions. Genomic DNA was removed from isolated total RNA by on-column DNase digestion. Then, cDNA was synthesized using 500 ng of total RNA and iScript™ reverse transcription Supermix (Bio-Rad), following the manufacturer’s instructions. Comparative real-time qPCR was performed in TaqMan® universal PCR master mix in triplicate using CFX96 Touch Real-Time PCR Detection System (Bio-Rad), according to the manufacturer’s protocol. TaqMan® probes and primers were manufactured by Applied Biosystems (Thermo Fisher Scientific): Mouse *Actb* (Mm02619580_g1), *B2m* (Mm00437762_m1), *Gapdh* (Mm99999915_g1), 18S (Mm03928990_g1), and *Tbp* (Mm00446971_m1) were used as endogenous controls for normalization of real-time qPCR. Relative expression was calculated using the comparative ∆∆Ct method.

**Western blot analysis**

Cell lysates were analyzed by western blotting using standard methods and indicated antibodies. Briefly, mouse MPCs were grown in complete culture media: DMEM supplemented with 10% FBS (Gibco) and antibiotics (penicillin (100 U/ml) and streptomycin (100 µg/ml, Thermo Fisher Scientific). Cells were seeded at 200,000 cells/well in 6-well tissue culture plates containing 2 ml/well of complete culture media. Cells were lysed in cell lysis buffer (50 mM Tris (pH 8.0), 150 mM NaCl, 1% IGEPAL, 0.5% sodium deoxycholate, 0.1% sodium disodium sulfate, 1× protease inhibitor cocktail, and 1× phosphatase inhibitor cocktail). Cell lysates were cleared by centrifugation at 20,000×g for 20 min at 4 °C after brief sonication. Soluble cell lysates were mixed with SDS-PAGE sample buffer (50mM Tris, pH 6.8, 2% SDS, 10% glycerol, 0.1% bromophenol blue, and 100mM dithiothreitol) and denatured at 95°C for 5 min. Proteins were resolved in 4–15% mini-PROTEAN precast gels (Bio-Rad) at 150V for 50~60 min. The resolved proteins were transferred to the PVDF membrane using the iBLOT2 dry blotting system (Thermo Fisher Scientific) following the manufacturer's instructions. Membranes were then immersed in 5% bovine serum albumin (BSA) in Tris-buffered saline (TBS) for 1h at room temperature (RT) to block non-specific binding of primary antibodies. Subsequently, Membranes were probed with the following primary antibodies: β-Actin (AC-15) mouse monoclonal antibody (A5441, Sigma-Aldrich), histone H3 (D1H2) XP® rabbit monoclonal antibody (4499, Cell Signaling Technology). Secondary antibodies were from Bio-Rad. After incubating NC membranes with primary antibodies for 16 h at 4°C, membranes were washed in TBS-T (1xTBS containing 0.5% Tween-20) three times for 5 min each at RT. Protein bands were visualized by incubating membranes for 1h at RT with secondary antibodies. The membranes were washed in TBS-T three times for 5 min each at RT before scanning with ChemiDoc Imaging Systems (Bio-Rad).

**Immunofluorescence staining of collagen ECM**

Cells were plated in glass bottom 2-well slide chambers and treated with DMSO (vehicle control) or PLOD2i compound refreshing media every 3 days for 4 weeks. At 4 weeks, cells were fixed with 4% PFA for 10 min and washed with 1x PBS three times for 5 min each at RT. Cells were blocked in 1% BSA and 0.02% Tween-20 in PBS. Cells were washed with 1x PBS three times and incubated with primary collagen α-1(I) chain carboxy-telopeptide antibody (clone LF-68, ENH018_FP, Kerafast) with 1% BSA in 1x PBS for 1h at RT. After washing with PBS, slides were incubated with fluorescence-conjugated secondary antibodies for 1h at RT (Alexa Fluor 555 donkey anti-rabbit IgG (A31572, Invitrogen). Following washing with 0.1% Triton X-100 in PBS, cells were stained for nucleus with Hoechst 33342 (H3570, Invitrogen) for 10 min at RT and washed two times with 0.1% TX-100 in PBS before imaging.

**Osteogenic differentiation and staining**

Cells were plated (50,000 cells/well) in 24-well tissue culture plates and cultured to confluency in α-MEM supplemented with 10% FBS (Gemini Bio-Products) and antibiotics (penicillin (100 U/mL) and streptomycin (100 μg/mL, Thermo Fisher Scientific) at 37'C and 5 % CO_2_. Post-confluence, cells were further cultured in osteogenic media (50 μg/mL L-ascorbic acid and 10 mM β-glycerophosphate, all from Sigma), refreshing osteogenic media every 3 days. At 7 days in osteogenic culture, cells were fixed and stained for alkaline phosphatase using alkaline phosphatase kit (86C, Sigma) following the manufacturer's instruction. To visualize ECM mineralization, cells were washed once with 1x PBS and fixed with 4% paraformaldehyde for 10 minutes at RT. Fixed cells were washed with 1x PBS and stained with 2% Alizarin Red S solution (pH 4.2) for 10 minutes at RT. Excess Alizarin Red S stain was removed by washing 5 times with distilled water. Cells were air-dried in the dark before imaging.

**Supplementary figures & legends:**

**
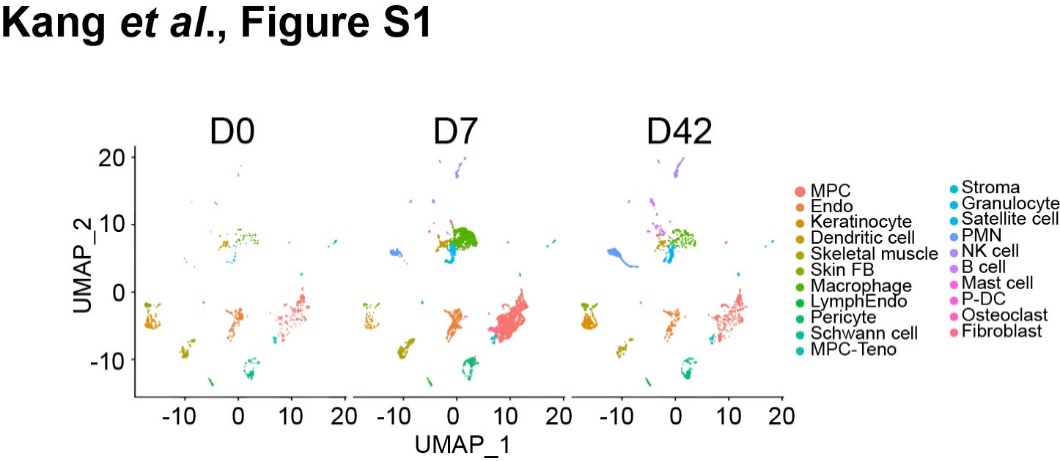
**

| **Figure S1.** UMAP cluster and cluster definitions of scRNA-seq at day 0, 7, and 42 post-BT injury. |
| --- |

**
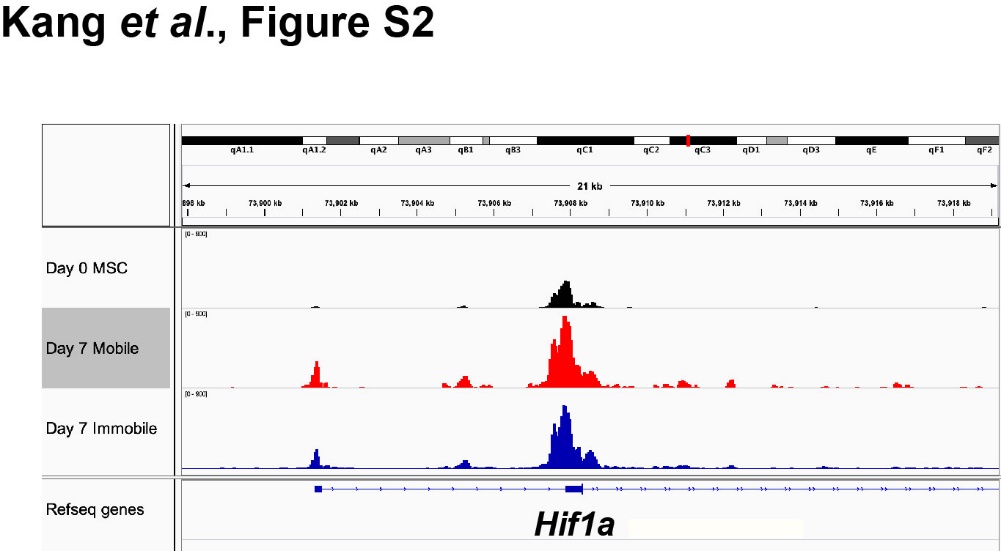
**

| **Figure S2. Chromatin opening of promoter region of HIf1a locus.** snATAC-seq peaks of the Hif1a promoter in MPC at day 7 post-injury (middle track; red) is more open compared to day 0 (top track; black) and day 7 post-injury/immobilization (bottom track; blue). |
| --- |

**
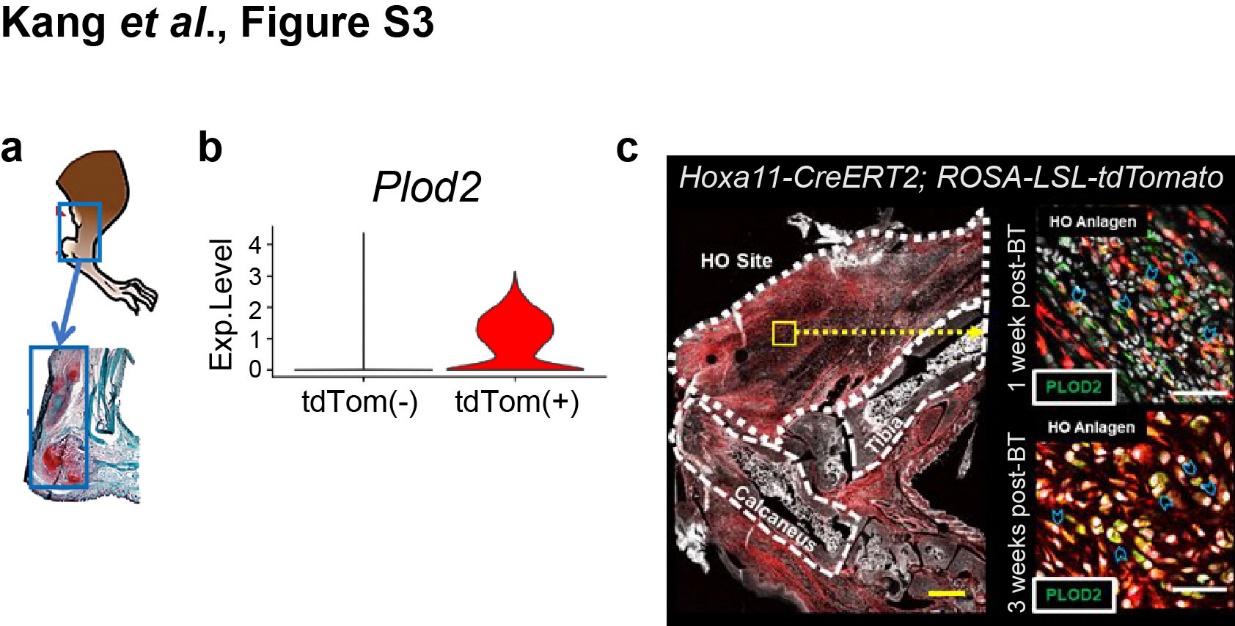
**

| **Figure S3. PLOD2 expression at the HO site.** (A) Schematic representation of HO induction by Achilles tenotomy following dorsal burn. (B) Expression of Plod2 in MPC cluster. (C) Representative images of immnofluorescent staining of injured limb tissue sections for PLOD2 protein level in PDGFRa (+) MPCs. at the HO site. |
| --- |

**
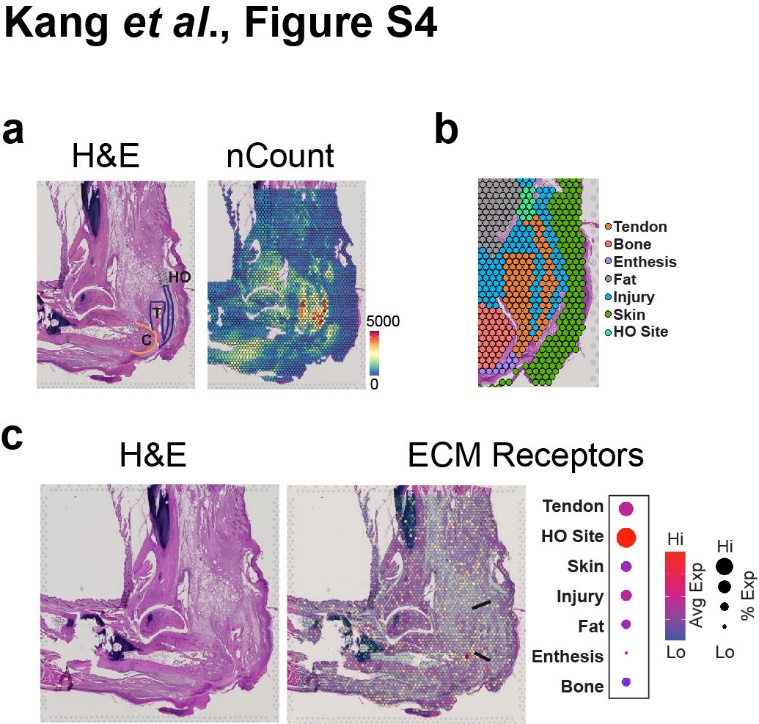
**

| **Figure S4.** (a) H&E stain of tenotomy injury site at 7 days post-BT, with posterior border of calcaneus (orange, “C”) and borders of Achilles tendon (blue, “T”) labeled. Area of HO formation at the end of the Achilles, where the tenotomy was performed, is boxed in green and labeled “HO". Box indicates zone of interest (left), with a zoomed-in view (middle) and feature overlay showing number of transcripts per spot (right). (b) SpatialFeaturePlot. (c) H&E stain of sample of tenotomy injury site 7 days post-BT (left). SpatialFeaturePlot (middle) and dot plot (right) showing expression of ECM receptors. Black lines indicate tendon ends. |
| --- |

**
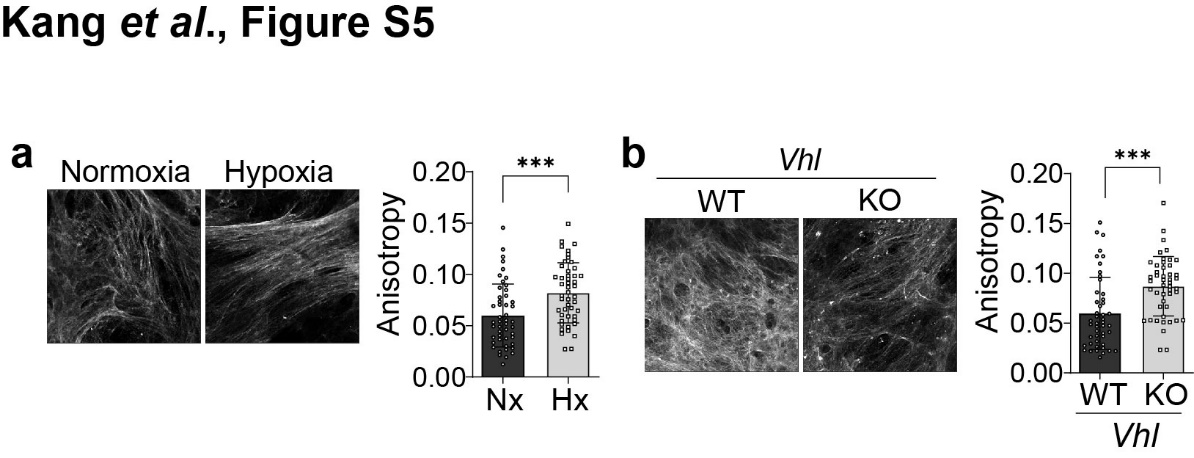
**

| **Figure S5. HIF-1α activation stimulates collagen fiber alignment.** (A) MPCs were cultured in normoxic or hypoxic condition. Collagen deposited after 4-week stimulation was immunostained with a collagen α1[I] C-telopeptide antibody. Graph shows quantification of anisotropy. Error bars represent mean ± SD. ***P < 0.001. Mann-Whitney unpaired t-test, two-tailed (n=48). (B) *Vhl* KO and WT control MPCs were cultured in normoxic condition. Graph shows quantification of anisotropy. Error bars represent mean ± SD. ***P < 0.001. Mann-Whitney unpaired t-test, two-tailed (n=47). |
| --- |

| **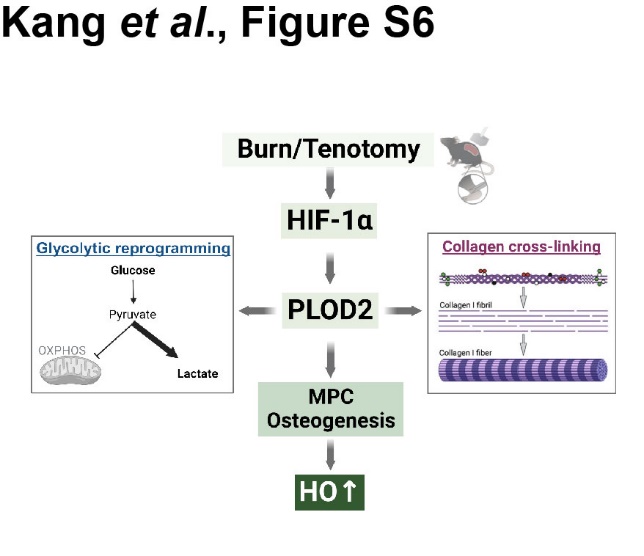**  **Figure S6. Summary model of HIF-1α action in HO**. (Created with BioRender.com) |
| --- |

**Table S1. Gene lists for spatial transcriptomics analysis**


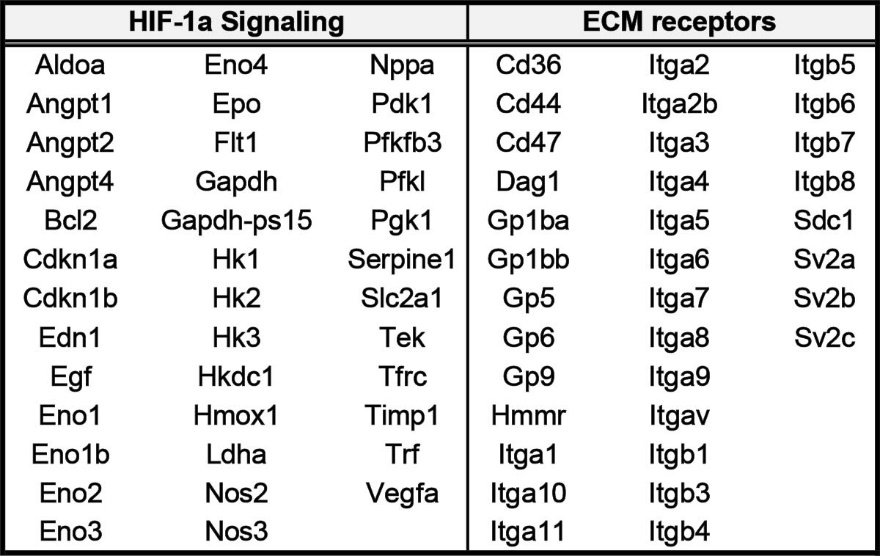

Supplement: Supplementary file 1 — Supplementary information [file 41413_2024_320_MOESM1_ESM.docx]
